# Genes with a large intronic burden show greater evolutionary conservation on the protein level

**DOI:** 10.1186/1471-2148-14-50

**Published:** 2014-03-16

**Authors:** Olga Gorlova, Alexey Fedorov, Christopher Logothetis, Christopher Amos, Ivan Gorlov

**Affiliations:** 1 Department of Community and Family Medicine, Geisel School of Medicine, Dartmouth College, Lebanon 03766, NH, USA; 2 University of Toledo, Toledo 43606, OH, USA; 3 Department of Genitourinary Medical Oncology, The University of Texas MD Anderson Cancer Center, Houston 77030, TX, USA; 4 Center for Genomic Medicine Department of Community and Family Medicine, Geisel School of Medicine, Dartmouth College, 46 Centerra Parkway, Suite 330, Lebanon 03766, NH, USA

**Keywords:** Exon/intron structure, Intronic burden, Evolutionary conservation

## Abstract

**Background:**

The existence of introns in eukaryotic genes is believed to provide an evolutionary advantage by increasing protein diversity through exon shuffling and alternative splicing. However, this eukaryotic feature is associated with the necessity of exclusion of intronic sequences, which requires considerable energy expenditure and can lead to splicing errors. The relationship between intronic burden and evolution is poorly understood. The goal of this study was to analyze the relationship between the intronic burden and the level of evolutionary conservation of the gene.

**Results:**

We found a positive correlation between the level of evolutionary conservation of a gene and its intronic burden. The level of evolutionary conservation was estimated using the conservation index (CI). The CI value was determined on the basis of the most distant ortholog of the human protein sequence and ranged from 0 (the gene was unique to the human genome) to 9 (an ortholog of the human gene was detected in plants). In multivariable model, both the number of introns and total intron size remained significant predictors of CI. We also found that the number of alternative splice variants was positively correlated with CI.

The expression level of a gene was negatively correlated with the number of introns and total size of intronic region. Genes with a greater intronic burden had lower density of missense and nonsense mutations in the coding regions of the gene, which suggests that they are under a stronger pressure from purifying selection.

**Conclusions:**

We identified a positive association between intronic burden and CI. One of the possible explanations of this is the idea of a cost-benefits balance. Evolutionarily conserved (functionally important) genes can “afford” the negative consequences of maintaining multiple introns because these consequences are outweighed by the benefit of maintaining the gene. Evolutionarily conserved and functionally important genes may use introns to create novel splice variants to tune the gene function to developmental stage and tissue type.

## Background

The division of genes into introns and exons is a hallmark of eukaryotic evolution. This division is believed to be evolutionarily beneficial because it allows the production of multiple proteins from the same gene through alternative splicing and may accelerate the creation of novel proteins through exon shuffling [1-4].

However, little is known about the forces that influence the exon/intron structure of genes [5-9]. Several biologically important characteristics correlated with intronic burden have been identified. For example, highly expressed genes tend to have shorter introns [10]. Similarly, a study of 391 genes from 19 eukaryotic species conducted by Carmel et al. [11] demonstrated that the probability of intronic gains is positively correlated with the level of evolutionary conservation of the gene. However, a whole-genome assessment of the association between the number and length of introns and the level of evolutionary conservation has not yet been conducted. We sought to determine whether evolutionary conservation was correlated with intronic load in a whole-genome analysis.

## Results and discussion

### Variation in number of introns among human genes

**Figure 1:**
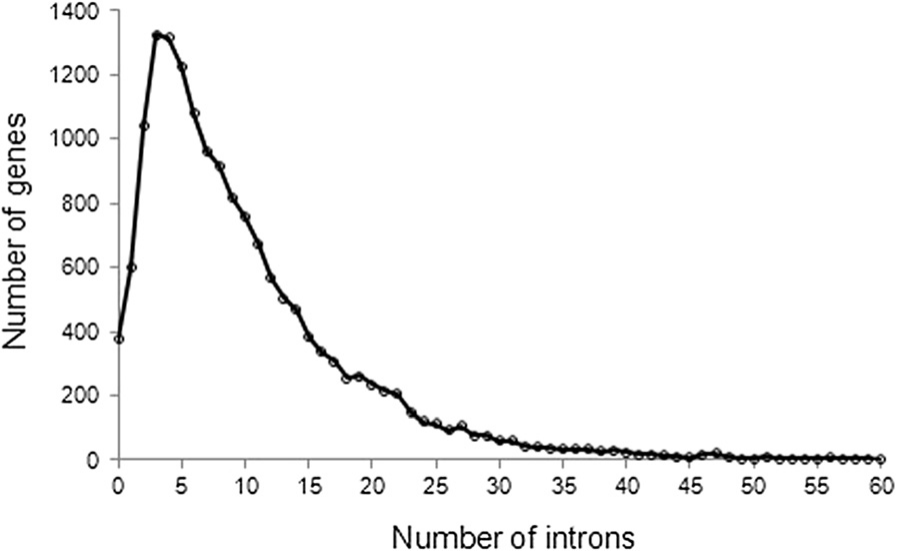
The distribution of the number of introns in the human genome.

There is significant variation in the number of introns in human genes (Figure 1). More than 600 human genes are intronless [12]. On the other side of the distribution, the *TTN* gene has more than 300 introns. The average number of introns per human gene is 8–9 [5]. The proportion of genes with small numbers of introns (0, 1, and 2) is relatively low (2%, 4%, and 6%, respectively). Genes with 3 to 6 introns are most common and comprise more than 30% of human genes. Genes with a larger number of introns are comparatively rare; genes with more than 30 introns comprise less than 5% of the genome.

### Association between the conservation index (CI), number of introns, and total intron size

**Figure 2:**
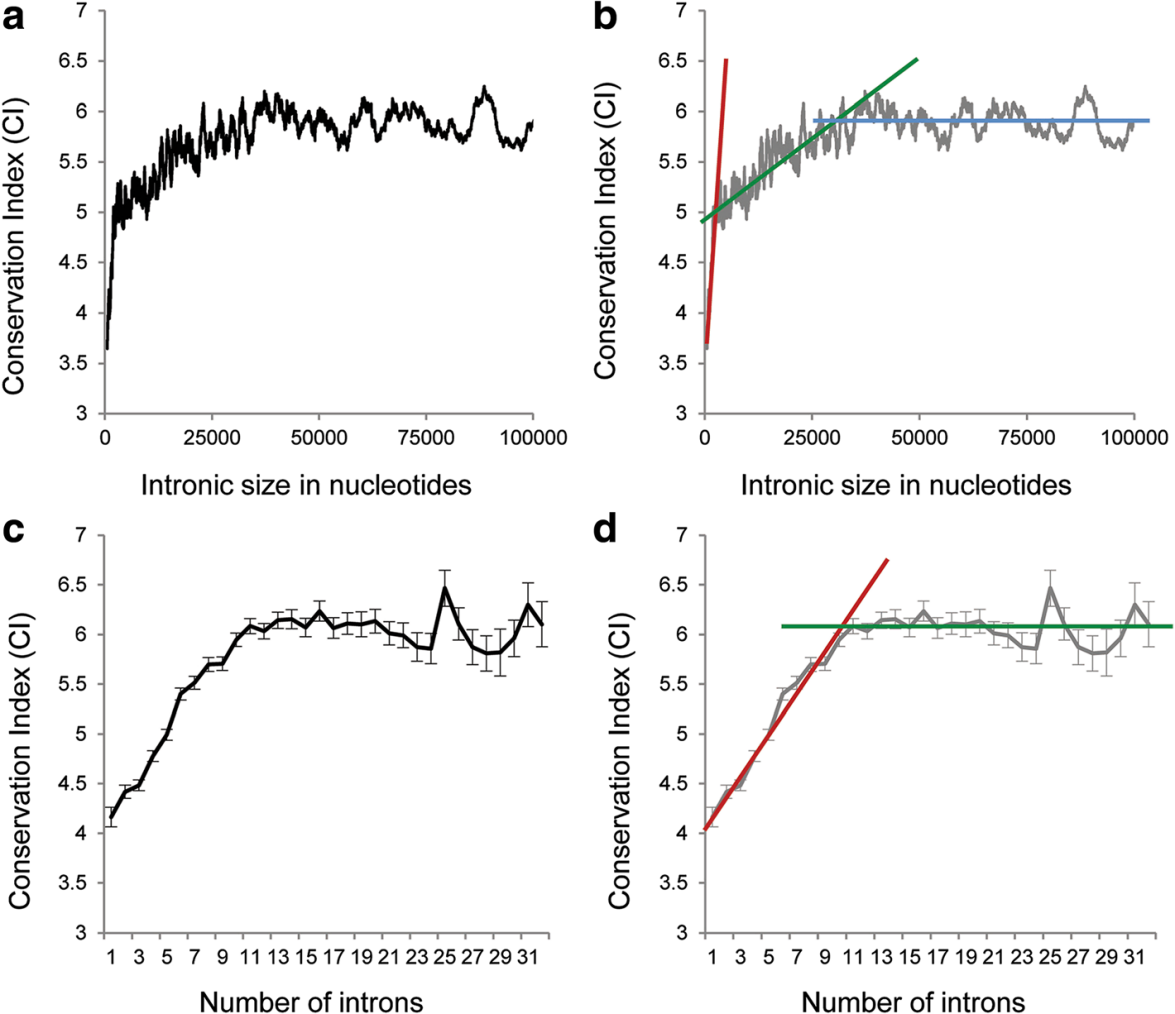
**The association between the conservation index (CI) and (a, b) the total intron size; (c, d) the number of introns.** Colored lines mark different segments of the curves.

There was a significant positive correlation between CI and the size of the intronic region (only genes with available CI were included in the analysis) (Spearman rank R = 0.06, N = 16,194, P < 10^−6^). Figure 2(a) and (b) detail the association between the total intron length and CI. Although there was a positive association between the total intron length and CI, the corresponding curve consists of several segments of different slope. We used segmented linear regression analysis to define the curve’s segments. The breakpoints were selected by maximizing the global R^2^ against a single segment model and penalizing for higher numbers of segments [13]. As a result, the association curve between the total intron length and CI was divided into three segments (Figure 2(b)). Segment 1 encompasses genes with the smaller intron lengths (18 nucleotides to 3 kb). The correlation between CI and the total intron length for this segment was Spearman rank R = 0.23 (N = 1,420, P < 10^−6^). In the second segment (genes with the total intron length of 3 to 30 kb), the correlation coefficient between CI and the intronic length was Spearman rank R = 0.10 (N = 6,642, P < 10^−6^). For the third segment (genes with intron length >30 kb), the correlation between CI and the intron size was not significant (Spearman rank R = −0.02, N = 8,189, P < 0.08).

We also observed a significant positive correlation between the number of introns in a gene and its CI (Spearman rank R = 0.16, N = 16,249, P < 10^−6^). Figure 2(c) and (d) show the relationship between the number of introns in a gene and the gene’s CI. On the basis of segmented regression analysis, the curve of this relationship was divided into two segments: the first segment of genes with 0 to 10 introns showed a linear positive correlation, and the second segment of genes with ≥ 11 introns showed a plateau in terms of correlation.

We used a multiple linear regression model to estimate the independent effects of the total intron length and the number of introns as predictors of CI. We analyzed only the genes that satisfied two conditions: 10 or fewer introns and total intron size between 3 and 30 kb (too few genes had a total intron size less than 3 kb). For this subset of the data the assumption of linearity is justified. We also checked the subset for independence of the errors, constant variance of the errors and normality of the distribution of the errors. Because all the conditions were met, multiple regression was applied to the data to assess whether the number of introns and the total intronic length are independent predictors of CI. In the multiple regression model including number of introns and total intron size both regression coefficients were significant: b_noi_ = 0.11, N = 1,420, P < 0.00003; b_til_ = 0.21, N = 1,420, P < 10^−6^. These results suggest that both the number of introns and the total intron size are independent predictors of CI for a subset of the data we have used.

### Intronic burden and gene expression

**Figure 3:**
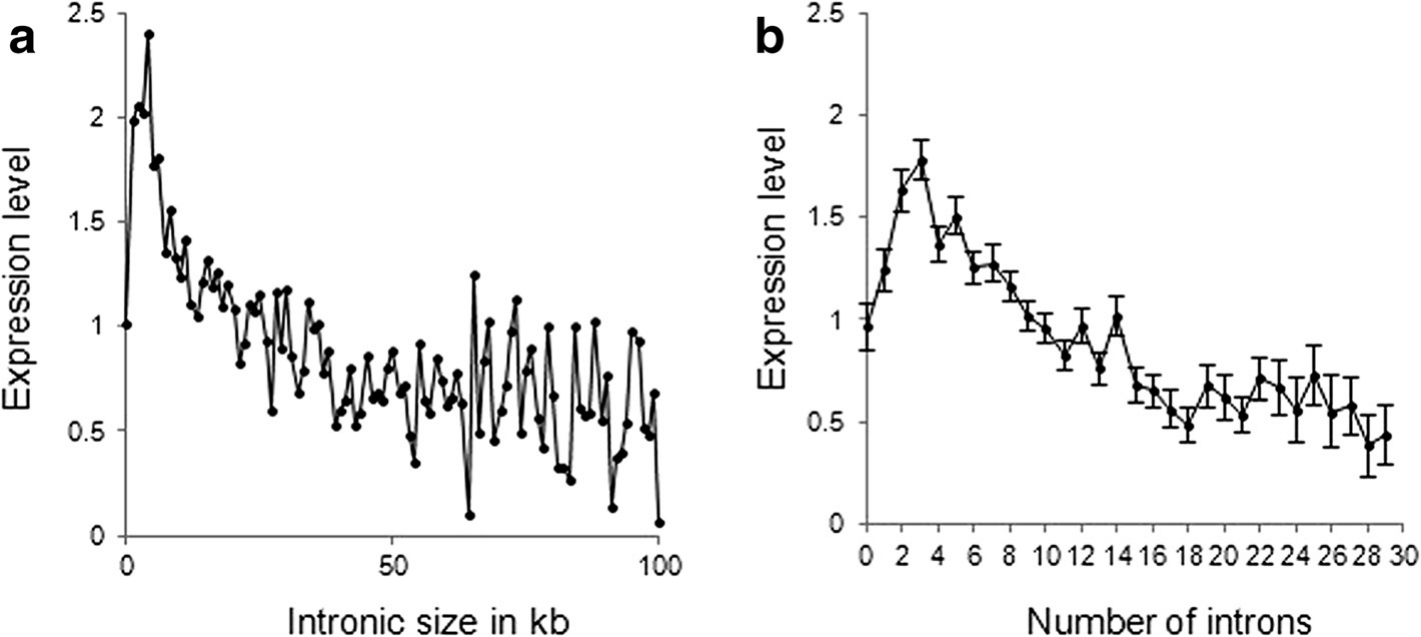
**The association between the mean gene expression levels in diverse normal human tissues and the total intron size (panel a) and number of introns (panel b).** Vertical lines on the right panel are standard errors of mean. Expression level is shown in Z-scores.

Figure 3(a) shows an association between the total intron lengths of 20,156 human genes and their mean gene expression levels in 10 normal human tissues [14]. The expression of the genes with small total intron size (less than 1 kb) was relatively low and increased markedly until the total intron size reached 5 kb. For the genes with the total intron lengths greater than 5 kb, there was a negative association with expression. A similar curve described the relationship between gene expression and the number of introns (Figure 3(b)). Intronless genes and those with a single intron had relatively low expression levels. The highest expression levels were observed in the group of genes with three introns, and then the average expression level decreased as the number of introns increased. The negative correlation between gene expression and intronic burden observed in this study supports the hypothesis that introns have a biological cost. It is known that transcription of introns is associated with considerable energy expenditures [15]. Similarly, splicing multiple introns out of mRNA is biologically expensive in terms of both energy expended and risk of splicing errors [16,17]. Larger introns are more prone to splicing errors [18]. Because having introns is associated with obvious biological disadvantages, it is logical to assume that only functionally important genes can support the burden of a large number of introns (or large total intron size).

The cost-benefit hypothesis predicts that genes with a large intronic burden are more functionally important than genes with a small intronic burden. A gene’s level of evolutionary conservation reflects its functional significance, and conserved genes are more likely to be involved in basic biological functions [19,20]. However, evolutionary conservation of a gene reflects not only its functional significance but also its evolutionary history, which is usually unknown. To further assess the functional significance of the genes, we used data on the density of functional polymorphisms. Genes with low functional importance accumulate functional (protein-changing) polymorphisms more easily [21,22]. Using dbSNP data, we found that the number of synonymous single nucleotide polymorphisms (SNPs) per codon did not correlate with CI (R = 0.01, N = 16,194, P = 0.18), whereas the number of non-synonymous SNPs and stop-gained SNPs was negatively correlated with CI (R = −0.1, n = 16,194, P < 10^−6^ and R = −0.06, n = 16,194, P < 10^−6^, respectively). These findings are consistent with the idea that the density of potentially functional polymorphisms can be used as a measure of the functional importance of a gene. We used the ratio of missense to synonymous substitutions (MIS/SYN) and the ratio of nonsense to synonymous substitutions (NON/SYN) to assess the strength of purifying selection on a gene. Strength of purifying selection can be used as a measure of functional importance of the gene under the assumption that more important genes are less tolerant to missense and nonsense substitutions. We noted a significant negative correlation between MIS/SYN and the number of introns (R = −0.05, n = 3,363, P = 0.006). The correlation between the number of introns and NON/SYN was also negative and significant (R = −0.26, n = 3,363, P < 10^−6^). The correlation between MIS/SYN and the total intron length was negative but did not reach statistical significance (R = −0.03, n = 3,363, P = 0.11), whereas the correlation between NON/SYN and the total intron length was significant (R = −0.13, n = 3,363, P < 10^−6^). These results support the idea that genes with a large total intron size and/or multiple introns tend to be more functionally important than the genes with smaller intronic loads as manifested in lower density of missense and nonsense substitutions.

We assume that functionally important genes may have more and larger introns because they can better “afford” a larger intronic burden than can less important genes. The other possible explanation for the maintenance of introns in functionally important genes may be related to alternative splicing. Up to 90% of human genes undergo alternative splicing [23]. Even if the majority of rare splice variants are the products of splicing errors [16], many of the alternative splice variants are functional [24-26]. Furthermore, exon boundaries often correspond to functional domains [27,28]. Therefore, it is conceivable that a larger number of introns enables functionally important genes to use alternative splicing to adjust and modify their functions on the basis of developmental stage or tissue type. We found a significant positive correlation between the number of splice variants and the CI of genes (R = 0.07, N = 15.819, P < 10^−6^). The association remained significant after controlling for the number of introns or the the total intron length (P = 0.0001) and suggests that functionally important genes are more likely to undergo alternative splicing.

Conversely, genes with a small number of introns (0–2) and a small total intron size (<3 kb) may be different from other genes. We found that the smallest genes in the human genome have the lowest CIs and low expression levels. These genes also have a higher density of non-synonymous and stop-gained SNPs than other genes (t-test values of 2.1 [P = 0.03] and 2.02 [P = 0.02] respectively), but they do not have a higher density of synonymous SNPs (t-test 0.2, df = 16,192, P = 0.87). This observation is consistent with the results of the analysis conducted by Krylov at al. [29]. In their analysis of 7 complete genomes, the authors demonstrated that the propensity of gene loss was positively correlated with the rate of accumulation of nucleotide substitutions and negatively correlated with the gene’s expression level. The smallest genes may be enriched with young genes that have yet to develop an important biological function and thus cannot accumulate or maintain multiple introns. An alternative possibility is that the smallest genes are mostly dying genes that for some reason (e.g., environmental changes) became less functional, lost their introns, and started to accumulate non-synonymous and stop-gained mutations.

### Heterogeneity of genetic composition across intronic groups

Database for Annotation, Visualisation, and Integrated Discovery (DAVID) http://david.abcc.ncifcrf.gov/home.jsp was used to check if the top and bottom 5% of the genes in terms of intronic burden were enriched by gene categories. We found that the bottom 5% of the genes with lowest number of introns are enriched by G-protein coupled receptor genes (GPCRs) (Benjamini P = 6.8E-54). This enrichment, however, is unlikely to drive low CI in the group. We found no significant difference in CI between GPCRs and other genes: average CI was 4.01 ± 0.11 for GPCRs and 4.19 ± 0.09 for other genes, Mann–Whitney U Test, adjusted Z = 0.34, P = 0.73. We also did not notice a significant difference in expression levels between GPRCs and other genes (nonparametric Mann Whitney test = 0.56, P = 0.24). The top 5% of genes with largest number of introns are enriched by ATP-binding genes (P value for enrichment is 1.1E-40) and cytoskeleton-associated genes (P value for enrichment is 2.2E-19).

We believe that heterogeneity of genetic composition is unlikely to be a major driving force behind the association between the intronic burden and CI. Firstly, the curve describing the relationship between intronic burden and CI is monotonic (Figure 2) suggesting that the intronic number rather than effect of the gene composition drives the association. For the middle part of the distribution gene enrichment is relatively low but we still see strong positive association between intronic burden and CI. Also for several gene families showing enrichment we found no evidence that the enrichment significantly contributes to the association.

## Conclusions

Our analysis demonstrated that evolutionarily conserved genes have a greater intronic burden. Previous research found a positive association between the level of evolutionary conservation and the size of the intronic region of a gene for a fraction of the human genes. We confirmed that association for the whole human genome. The results of our analysis also suggest that not only the total intronic length but also the number of introns is an independent predictor of the level of evolutionary conservation of a gene.

Using the latest and most precise estimates of gene expression levels, we demonstrated a negative association between the intronic burden and the level of expression. Similar to the above analysis, this investigation showed that both the total intronic length and the number of introns independently predict expression level. We found that the genes with the lowest intronic burden are different from most other human genes, suggesting that they could be evolutionarily young (which is why they tend to have a lower conservation index) and have yet to acquire an indispensable biological function.

In conclusion, the problem of evolutionary advantages (or disadvantages) of introns is complex. Having multiple introns is obviously associated with a burden in terms of energy and resources: a cell needs first to transcribe all introns, then to remove them. It has been shown that splicing is often associated with errors that produce abnormal product and may have a negative effect on cell survival [1]. On the other hand, having multiple introns provides an opportunity for alternative splicing which can be associated with distinct and important biological functions [2]. It is not clear if by alternative splicing the cell tries to make use of something already available, or alternative splicing is a driver of intronic additions. It is also possible that introns are gained more often in important genes because they have higher expression levels and more open chromatin structure. Additionally it is possible that the disadvantage of an intron gain is less in important compared to not-so-important genes. We demonstrated that both the total size of intronic sequences and the total number of introns are independent predictors of the level of evolutionary conservation. Our findings raise an interesting possibility that intronic burden could be used as a predictor of the functional importance of a gene.

## Methods

We used CI as a measure of evolutionary conservation of the protein sequence. CI values were assigned on the basis of the most distant ortholog of the human gene using data taken from the HomoloGene database [30]. HomoloGene provides a list of orthologs detected in 20 completely sequenced genomes: *M. oryzae, M. mulatta, H. sapiens, P. troglodytes, C. lupus, B. taurus, M. musculus, R. norvegicus, G. gallus, D. rerio, D. melanogaster, A. gambiae, C. elegans, S. pombe, S. cerevisiae, K. lactis, E. gossypii, N. crassa, A. thaliana,* and *O. sativa.* These species were ranked on the basis of their evolutionary distance from humans. Because the estimated time of divergence between some of these species (e.g., *M. musculus* and *R. norvegicus*) and humans is essentially the same [31], we assumed that they marked the same time point in the evolutionary past. Also, not for all species divergence time data are available. As a result, 20 completely sequenced species provided only 10 divergence time points (Table 1). CI ranged from 0 (when a gene was unique to *H. sapiens*) to 9 (when a human ortholog was detected in any plant species). The approach we used was similar to the approach used by Domazet-Loso and Tautz [32], except we used alignments from HomoloGene [30,33], whereas Domazet-Loso and Tautz performed their own protein sequence alignments.

**Table 1:** Conservation index (CI) scale

**An example of the most distant species with detectable human ortholog**	**Phylogenic group**	**Divergence time (million years)**	**Reference**	**CI**
*Homo sapiens*	Unique to humans			0
*Pan troglodytes*	Great apes	6	[31]	1
*Macaca mullata*	Primates	35	[31]	2
*Rattus norvegicus*	Rodents	90	[31]	3
*Gallus gallus*	Birds	310	[34]	4
*Danio rerio*	Fishes	400	[34]	5
*Anopheles gambiae*	Insects	600	[35]	6
*Caenorhabditis elegans*	Worms	700	[35]	7
*Schizosaccharomyces pombe*	Fungi	800	[35]	8
*Oryza sativa*	Plants	900	[35]	9

Data on the exon/intron structure of genes, including the number of alternatively spliced isoforms, were obtained from the Exon-Intron Database. This database was created and is curated by one of the authors (A.F.) [36], is based on NCBI Gene Bank data, and allows large-scale computational examination of exon/intron structure. We assessed gene expression levels using data from the RNA-Seq Atlas database [14]. This database provides estimates of gene expression levels across 10 human tissues (colon, heart, hypothalamus, kidney, liver, lung, ovary, skeletal muscle, spleen, and testes) and is based on RNA sequencing, which provides a less biased estimate of gene expression than probe-based technologies. As previously published, Z-scores were used as quantitative measures of gene expression [14]. In brief, Z-score is a measure of the expression of an individual gene to the gene’s relative to the expression distribution in a reference population. The reference distribution is based on the distribution of the expression of all genes in all tissues. The expression value of a given gene in given tissue (Z-score) represents the number of standard deviations away from the mean of expression in the reference population.

To assess the correlation between the density of functional polymorphisms (missense and nonsense mutations) and intronic burden, we used the dbSNP database. The density of functional polymorphisms in a given gene was computed by dividing the total number of reported missense and nonsense mutations by the size of the coding region. Only validated SNPs were used for the analysis. We used the number of SNPs per codon as a measure of the SNP density. The nonparametric Spearman correlation coefficient was used to assess the correlation between the size and number of introns and the CI. To identify segments of linearity we used R^2^-based method [13]. Statistical analysis was done using STATA software (version 10, StataCorp LP, College Station, TX).

## Competing interests

The authors declare that they have no competing interests.

## Authors’ contributions

OG participated in the design of the study and performed the statistical analysis. AF participated in statistical analysis and data retrieving. CL participated in the design of the study. CA participated in the design of the study and drafted the manuscript. IG conceived of the study, and participated in its design and coordination. All authors read and approved the final manuscript.
